# Effectiveness of an individualized program of muscular strength and endurance with aerobic training for improving germ cell cancer-related fatigue in men undergoing chemotherapy: EFICATEST study protocol for a randomized controlled trial

**DOI:** 10.1186/s13063-015-1143-x

**Published:** 2016-01-05

**Authors:** Antonio Ignacio Cuesta-Vargas, Francisco Carabantes, Zaira Caracuel, Inmaculada Conejo, Emilio Alba

**Affiliations:** Department of Physiotherapy, Faculty of Health Sciences, Instituto de Investigacion de Biomedicina de Malaga (IBIMA), Universidad de Malaga, Málaga, Spain; School of Clinical Science, Faculty of Health, Queensland University of Technology, Kelvin Grove, QLD Australia; Department of Medical Oncology, Carlos Haya Regional University Hospital, Málaga, Spain; Department of Cellular Biology, Genetics and Physiology, Faculty of Sciences, Universidad de Malaga, Málaga, Spain

**Keywords:** Testicular cancer, Germ cell cancer, Physical exercise, Cancer-related fatigue, Chemotherapy, Clinical rules, Metabolomics

## Abstract

**Background:**

Patients with testicular germ cell cancer (GCC) have a high cure rate; however, cancer-related fatigue is the most common complication among patients with GCC undergoing treatment with chemotherapy. Although exercise is widely recommended, information about the physio-pathological effects of cancer therapy on skeletal muscle is very limited. Our aim is to evaluate the effects of an individualized program of muscular strength and endurance with aerobic training on cancer-related fatigue.

**Methods/Design:**

The present study is a randomized controlled trial comparing an individualized program of muscular strength and endurance with aerobic training compared to a control group. We will conduct this trial in patients undergoing chemotherapy, recruited by the Department of Oncology of Virgen de la Victoria Hospital (Málaga). Patients will be included and evaluated before the first cycle of chemotherapy and assigned randomly to the experimental or control group. Cancer-related fatigue, physical condition and biological samples will be measured at the beginning and at the end of an 8-week intervention by the same evaluator, who will be unaware of the allocation of participants to each group. Furthermore, there will be monitoring for 6 months (24 weeks) after training for all outcome variables.

**Discussion:**

This study hopes to offer patients with GCC an individualized exercise program with aerobic training for cancer-related fatigue. Such a scheme, if beneficial, could be implemented successfully within public health.

**Trial registration:**

ClinicalTrials.gov Identifier: NCT02433197. Date of registration: 13 April 2015.

## Background

Testicular germ cell cancer (GCC) represents 1 % of all human cancers and its incidence has increased by 1 % over the last 50 years [1, 2]. In young men, it represents one of the most malignant forms and is the second commonest cause of cancer-related death [1, 2]. Testicular tumor cell growth constitutes the majority of testicular malignancies [2]. GCC patients have a high cure rate, and are sensitive to radiation and chemotherapy, but 5 % of patients develop resistance to treatment.

Impaired muscle function and cancer-related fatigue (CRF) are two of the most common complications among patients in additiounder chemotherapy [3]. Moreover, early, localized muscular fatigue and severe deconditioning are common observations in clinical practice involving GCC patients undergoing chemotherapy [4]. The cause of this muscle deconditioning is unknown and although the effects of antineoplastic drugs have been described in some detail [4, 5], the way these effects may affect the phenomenon of CRF is poorly described. With growing interest in the duality oncology-physical exercise, many studies are now focused on the implementation of physical exercise as a complementary intervention to anti-cancer therapy [6]. Although exercise is widely recommended, information about the physio-pathological effects of cancer therapy on skeletal musculature is very limited [7]. Severe and chronic fatigue has been found in 30 % of long-term GCC survivors many years after the end of primary treatment [8]. The phenomenon known as “cancer-related fatigue” is characterized by being more severe, more distressing and having less chance of relief through rest than in the general population [9]. Although there are several questionnaires to assess CRF [10], a complementary method such as semi-structured [11] could be used.

Treatment with aerobic training in association with education has been shown to induce clinically very significant improvements in breast CRF [4]. In addition, a program of strength and muscular endurance training has been shown to attenuate the reductions in fiber size and strength in healthy subjects and patients [4, 12].

Metabolomics is an emerging discipline, defined as a multidisciplinary science that requires cooperation between chemists, biologists and computer scientists. Metabolome is the term used to refer to the complete inventory of small molecules, non-protein compounds, such metabolic intermediates, ATP, fatty acids, glucose, cholesterol, hormones, and other signaling molecules, and secondary metabolites found in a biological sample [13, 14]. As such, the metabolome changes continuously, depending on the activation and interaction of the various metabolic pathways inside the cell. It also reflects the phenotype that can be used to interfere gene function. Although genomics and proteomics can provide important information on expected function, metabolomics provides an immediate snapshot of all biological functions that reflect current events at a specific time [15].

The primary objective of the study is to investigate the short- and long-term (6 months) effects of an individualized program of muscular strength and endurance with aerobic training to improve CRF. A secondary goal is to examine the tolerance of muscular endurance training started on day 1 of the course of cancer treatment on muscle deterioration. Furthermore, the effect of the program on metabolomic level and on various fitness and quality-of-life parameters will be analyzed to try and establish rules of clinical prediction by multivariate regressions. The hypothesis of our study is that a program of muscular strength and endurance with aerobic training to improve CRF can be designed.

## Methods/Design

### Design and participants

The present study is a randomized controlled clinical trial conforming to Consolidated Standards of Reporting Trials (CONSORT) guidelines, comparing an individualized program of strength and muscular endurance with aerobic training versus a control group. All variables will be measured at the beginning and the end of the 8-week intervention by the same evaluator, who will be unaware of the allocation of participants to each group. Furthermore, there will be monitoring for 6 months (24 weeks) after training for all outcome variables. Evaluations will be completed in the Area Health Patronage of Sports, Torremolinos, Spain.

**Fig. 1:**
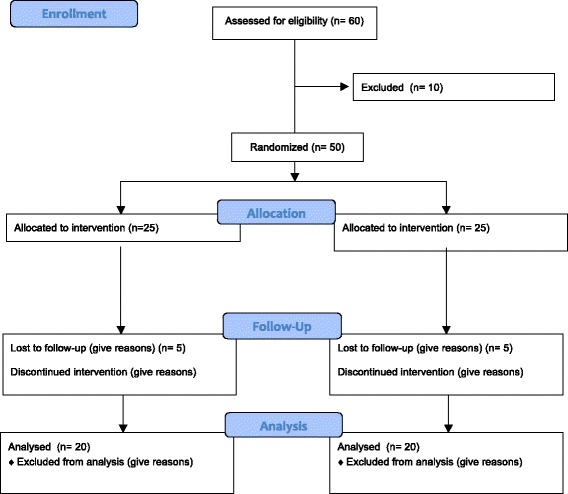
Flow chart of the study

GCC patients between 18 and 45 years who are due to undergo chemotherapy at the University Hospital of Málaga will be included in the study. The sample selection will be among those with performance status (PS) ≤1. Patients will be included and evaluated before the first cycle of chemotherapy and assigned to the experimental or control group randomly by using hidden envelopes. Potential participants will be contacted by the study coordinator and all doubts will be resolved before giving their written consent from each participant. The expected flowchart is shown in Fig. 1. The study had ethics approval from the Comité de Ética de la Investigación Provincial de Málaga (Consejería de Salud Servicio Andaluz de Salud, Spain). The principles of the Declaration of Helsinki are respected.

### Treatment

Antineoplastic therapy for GCC will consist of cisplatin 20 mg/m^2^ and etoposide 100 g/m^2^ daily for 5 days and bleomycin 15,000 IU/m^2^ weekly. Three cycles of this combination will be administered in 3-week cycles.

### Intervention

The intervention will be an 8-week individualized program of muscular strength and endurance with aerobic training, led by physiotherapists in groups of eight to ten participants. The 8-week program is a good balance between the minimum necessary to produce physiological improvements and the maximum to avoid drop-out. Each program will be individualized based on the evaluations of muscular strength and endurance as well as determination of the aerobic-anaerobic zone transition described in previous studies [16–19]. Sessions will be of 1 hour duration, three times a week. Each session will consist of 30 minutes of strength exercises and 20 minutes of continuous exercises in the aerobic-anaerobic zone transition.

Land-based exercises to improve muscular strength and endurance will follow the protocol described by Andersen and Aagaard [12]. In weeks 1 and 2, participants carry out 3 sets of 15 repetitions (reps) to become familiar with the exercises. From week 3 onwards, participants will perform 4 sets of 10 reps. If the participant can do more than 12 reps, the weight to be lifted will be increased. All exercises are carried out under supervision to ensure proper technique and adequate progression.

Aerobic training exercises will be performed using a cycloergometer [16]. In weeks 1 and 2, participants perform a low-level intervallic adaptation to adapt to the experience of fatigue during exercise. For the six remaining weeks, participants will be instructed to maintain a constant rhythm in the cardiac training prescribed based on an individual test [16] and their gradual adaptation to lactate tolerance [6]. If the participant experiences discomfort or severe fatigue during intense exercise, intensity will be reduced whilst maintaining the objective whenever possible. Participants will be encouraged to participate in all sessions and will be continually reminded of the importance of adherence in order to achieve the objectives.

Both groups, intervention and control, receive a printed decalogue based on common psychosocial issues for GCC survivors: having cancer and its implications, stress, distress, uncertainty, fear and anxiety, doubts about body image, family relations and future prospects [20]. The control group will be instructed to continue their current activities and to not objectively increase levels of physical activity performed during the 8-week intervention.

### Measurements

During the study, course participants will complete questionnaires and interviews, and tests of physical condition and sampling of tissues and cells will also be carried out. The primary outcome will be the change in CRF as measured by the QuickPIPER questionnaire and also general health and quality of life will be evaluated by validated scales, as well as an objective assessment of physical condition by a standardized and validated dynamometer test.

### Primary outcomes

The primary outcome will be fatigue, assessed by the revised CRF QuickPIPER scale [21], with the semi-structured interview [11] to define CRF phenotype.

### Secondary outcomes

Secondary outcome variables were distributed among the questionnaires, a physical condition test and biological samples.

Medical and demographic information will be collected about age, marital status, educational level and surgery for GCC. State of physical and mental health will be evaluated through the short form (SF-12) [22] and quality of life (EuroQoL-5D and EuroQoL-VAS) questionnaires [23]. The study of fatigue will be completed by the assessment of mood state by POMS (Profile of Mood States). It is possible to obtain an overall index of altered mood and seven partial measures: stress/anxiety, depression/rejection, anger/hostility, vigor/activity, fatigue/inertia and confusion/bewilderment [24].

A physical condition test will be included to assess isotonic peak strength of knee extension (quadriceps), knee flexion (hamstring), elbow extension (triceps brachii) and elbow flexion (biceps brachii) by one dynamometric measurement instrumented with Powertruck II of JTECH, following the protocol described by Daniels and Worthingham [25]. Hand grip strength will be evaluated by the hydraulic dynamometer JAMAR [26].

Biological samples are taken to determine the immunohistochemical, molecular and systemic changes.

Metabolomic analysis and systemic inflammation will be measured in 10-ml venous blood samples following clinical standards. Metabolomics can be used to systematically measure the population of biomarkers (metabolites), creating profiles among healthy individuals versus those with specific diseases [27]. Moreover, metabolomics may provide signs of a metabolic problem or injury with high precision and less cost than genomics, transcriptomics or proteomics and can, therefore, be a very suitable technique for generalized scientific research [28, 29]. Fluid metabolomic analysis is being used successfully in cancer [30–33]). Circulating plasma cytokines will be analyzed using antifreeze, ethylenediaminetetraacetate (EDTA). Plasma will be stored at −80 °C until further analysis. C-reactive protein (CRP), tumor necrosis factor alpha (TNF-α), interleukin (IL)-6 IL-18, IL-4 and IL-10 will be determined by ELISA (Enzyme-linked Immunosorbent Assay) as previously described [34]. Total cholesterol, high-density lipoprotein (HDL), low-density lipoprotein (LDL) cholesterol, triglycerides, glucose and insulin will be determined by standard laboratory procedures.

### Sample size

Version 3.1 G-Power was used to estimate the sample size and a minimum of 20 subjects per group (10 % loss) will be required “a priori” to have sufficient statistical power (80 %), alpha error (0.05) and a clinical effect Cohen’s *d* = 0.87 in the QuickPIPER scale of CRF [24].

### Statistical treatment

For analysis of the results, a database will be created using the information collected from participants’ notebooks (questionnaires, interviews, physical tests and biological tests). Intention-to-treat analysis of all participants will be developed. After the intervention phase, descriptive statistics are performed with measures of central tendency and dispersion of the study variables. This is followed by inferential analysis by difference values between the outcome variables in both groups. In addition, multiple regression analysis of potential predictors is carried out to determine clinical prediction rules for severe fatigue and other effects of cancer treatment. The size of intergroup effect will be calculated (Cohen’s *d*). An effect size <0.2 reflects a negligible difference, between ≥0.2 and ≤0.5 a small difference, between ≥0.5 and ≤0.8 a moderate difference, and ≥0.8 a large difference. A value of *p* <0.05 will be considered statistically significant. SPSS V 21.0 will be used in Mac version for data analysis.

## Discussion

The study results will be quickly applied in clinical practice guidelines thanks to the internal and external validity of the study, whether the hypothesis is accepted or not. Such a scheme, if beneficial, could be implemented within public health, after certain requirements are resolved with regard to resistance-training facilities, with instructors who are properly educated in order to train cancer patients undergoing chemotherapy. Patients with testicular GCC are offered an individualized program of muscular strength and endurance with aerobic training on CRF. In addition, regression analysis may open up new niche clinical studies on this question.

## Trial status

This study is not yet open for participant recruitment at the time of submission.

## Abbreviations

CRF: cancer-related fatigue
CRP: C-reactive protein
EDTA: ethylenediaminetetraacetate
ELISA: Enzyme-linked Immunosorbent Assay
GCC: germ cell cancer
HDL: high-density lipoprotein
IL: interleukin
LDL: low-density lipoprotein cholesterol
POMS: Profile of Mood States
PS: performance status
TNF-α: tumor necrosis factor alpha

## References

[1] Jemal A, Tiwari RC, Murray T, Ghafoor A, Samuels A, Ward E (2004). Cancer statistics. CA Cancer J Clin.

[2] American Cancer Society (2010). Cancer facts & figures.

[3] Adamsen L, Quist M, Andersen C, Moller T, Herrstedt J, Kronborg D (2009). Effect of a multimodal high intensity exercise intervention in cancer patients undergoing chemotherapy: randomised controlled trial. BMJ.

[4] Christensen JF, Jones LW, Tolver A, Jørgensen LW, Andersen JL, Adamsen L (2014). Safety and efficacy of resistance training in germ cell cancer patients undergoing chemotherapy: a randomized controlled trial. Br J Cancer.

[5] Christensen JF, Andersen JL, Adamsen L, Lindegaard B, Mackey AL, Nielsen RH (2011). Progressive resistance training and cancer testis (PROTRACT) – efficacy of resistance training on muscle function, morphology and inflammatory profile in testicular cancer patients undergoing chemotherapy: design of a randomized controlled trial. BMC Cancer.

[6] Cuesta-Vargas AI, Buchan J, Arroyo-Morales M (2014). A multimodal physiotherapy programme plus deep water running for improving cancer-related fatigue and quality of life in breast cancer survivors. Eur J Cancer Care.

[7] Galvão DA, Newton RU (2005). Review of exercise intervention studies in cancer patients. J Clin Oncol.

[8] Schultz PN, Klein MJ, Beck ML, Stava C, Sellin RV (2005). Breast cancer: relationship between menopausal symptoms, physiologic health effects of cancer treatment and physical constraints on quality of life in longterm survivors. J Clin Nurs.

[9] Holzner B, Kemmler G, Meraner V, Maislinger A, Kopp M, Bodner T (2003). Fatigue in ovarian carcinoma patients: a neglected issue?. Cancer.

[10] Minton O, Stone P (2009). A systematic review of the scales used for the measurement of cancer-related fatigue (CRF). Ann Oncol.

[11] Cella D, Peterman A, Passik S, Jacobsen P, Breitbart W (1998). Progress toward guidelines for the management of fatigue. Oncology (Williston Park).

[12] Andersen JL, Aagaard P (2000). Myosin heavy chain IIX overshoot in human skeletal muscle. Muscle Nerve.

[13] Nicholson JK, Lindon JC (2008). Systems biology: metabonomics. Nature.

[14] Deepinder F, Chowdary HT, Agarwal A (2007). Role of metabolomic analysis of biomarkers in the management of male infertility. Expert Rev Mol Diagn.

[15] Courant F, Antignac JP, Monteau F, Le Bizec B (2013). Metabolomics as a potential new approach for investigating human reproductive disorders. J Proteome Res.

[16] Cuesta-Vargas AI, Heywood S (2011). Aerobic fitness testing in chronic nonspecific low back pain: a comparison of deep water running with cycle ergometer. Am J Phys Med Rehabil.

[17] Cuesta-Vargas AI, García-Romero JC, Arroyo-Morales M, Diego-Acosta AM, Daly DJ (2011). Exercise, manual therapy, and education with or without high-intensity deep-water running for nonspecific chronic low back pain: a pragmatic randomized controlled trial. Am J Phys Med Rehabil.

[18] Cuesta-Vargas AI, García-Romero JC, Kuisma R (2009). Maximum and resting heart rate in treadmill and deep-water running in male international volleyball players. Int J Aquatic Res Educ.

[19] Cuesta-Vargas AI, Adams N, Salazar JA, Belles A, Hazañas S, Arroyo-Morales M (2012). Deep water running plus general practice for non-specific low back pain alone: randomised controlled trial. Clinical Rheumatol.

[20] Naumann F, Martin E, Philpott M, Smith C, Groff D, Battaglini C (2012). Can counselling add value to an exercise intervention for improving quality of life in breast cancer survivors? A feasibility study. J Support Oncol.

[21] Cuesta-Vargas AI, Férnandez-Lao C, Cantarero-Villanueva I, Castro-Sánchez AM, Fernández-de-Las Peñas C, Polley MJ (2013). Properties of the QuickPIPER: a shortened version of the PIPER Fatigue scale. Eur J Cancer Care (Engl).

[22] Vilagut G, Valderas JM, Ferrer M, Garin O, López-García E, Alonso J (2008). Interpretation of SF-36 and SF-12 questionnaires in Spain: physical and mental components. Med Clin.

[23] Jia H, Lubetkin EI (2008). Estimating EuroQoL EQ-5D scores from Population Healthy Days data. Med Decis Making.

[24] Cantarero-Villanueva I, Fernández-Lao C, Díaz-Rodríguez L, Cuesta-Vargas AI, Fernández-de-las-Peñas C, Piper BF (2014). The Piper Fatigue Scale-Revised: translation and psychometric evaluation in Spanish-speaking breast cancer survivors. Qual Life Res.

[25] Daniels L, Worthingham C (1995). Muscle testing: techniques of manual examination.

[26] Bellace JV, Healy D, Besser MP, Byron T, Hohman L (2000). Validity of the Dexter evaluation system’s Jamar dynamometer attachment for assessment of hand grip strength in a normal population. J Hand Ther.

[27] Tomlins AM, Foxall PJ, Lynch MJ, Parkinson J, Everett JR, Nicholson JK (1998). High resolution 1H NMR spectroscopic studies on dynamic biochemical processes in incubated human seminal fluid samples. Biochim Biophys Acta.

[28] Groenen PM, Engelke UF, Wevers RA, Hendriks JC, Eskes TK, Merkus HM (2004). High-resolution 1H NMR spectroscopy of amniotic fluids from spina bifida fetuses and controls. Eur J Obstet Gynecol Reprod Biol.

[29] Ellis DI, Goodacre R (2006). Metabolic fingerprinting in disease diagnosis: biomedical applications of infrared and Raman spectroscopy. Analyst.

[30] Deyati A, Younesi E, Hofmann-Apitius M, Novac N (2013). Challenges and opportunities for oncology biomarker discovery. Drug Discov Today.

[31] Buzatto AZ, de Sousa AC, Guedes SF, Cieslarová Z, Simionato AV (2014). Metabolomic investigation of human diseases biomarkers by CE and LC coupled to MS. Electrophoresis.

[32] Vermeersch KA, Styczynski MP (2013). Applications of metabolomics in cancer research. J Carcinog.

[33] Decelle EA, Cheng LL (2014). High-resolution magic angle spinning 1H MRS in prostate cancer. NMR Biomed.

[34] Lindegaard B, Hansen T, Hvid T, van Hall G, Plomgaard P, Ditlevsen S (2008). The effect of strength and endurance training on insulin sensitivity and fat distribution in human immunodeficiency virus-infected patients with lipodystrophy. J Clin Endocrinol Metab.

